# Cyber Child-to-Parent Violence and Child-to-Parent Violence: Bidirectional Trajectories and Associated Longitudinal Risk Factors

**DOI:** 10.3390/ejihpe15100190

**Published:** 2025-09-23

**Authors:** Sara Rodriguez-Gonzalez, Ainara Echezarraga, Joana Del Hoyo-Bilbao

**Affiliations:** Deusto Stress Research, Department of Psychology, Faculty of Health Science, University of Deusto, Av. de las Universidades, 24, 48007 Bilbao, Spain; a.echezarraga@deusto.es (A.E.); joana.delhoyo@deusto.es (J.D.H.-B.)

**Keywords:** cyber child-to-parent violence, child-to-parent violence, adolescence, family relationships, distress, substance abuse

## Abstract

Offline forms of violence are evolving into their online counterparts. The aim of this study was to examine cyber child-to-parent violence by (1) analyzing its bidirectional relationships with offline CPV and distress, (2) identifying individual risk factors (distress and substance abuse) and family-related risk factors (exposure to family violence, parental ineffectiveness, parental impulsivity, and punitive discipline) regarding Cyber-CPV, (3) examining individual and family-related risk factors for CPV and (4) evaluating the moderating role of substance abuse in the relationship between distress and Cyber-CPV. The study sample consisted of 1034 adolescents (*M*_age_ = 15.05; *SD* = 1.53), who completed the measures at two time points, six months apart. The results show bidirectional relationships between Cyber-CPV and distress. Furthermore, CPV significantly predicted the occurrence of Cyber-CPV over time. Moreover, substance abuse predicted Cyber-CPV and moderated the association between distress and Cyber-CPV; this association was strengthened under high levels of substance abuse. Additionally, both substance abuse and punitive discipline predicted CPV. This study highlights the predominant role of individual factors in Cyber-CPV, and it provides pioneering insights into the related variables of this emerging form of intrafamilial violence mediated by digital technologies, thus laying the groundwork for future research.

## 1. Introduction

Child-to-Parent Violence (CPV) refers to physical, psychological, and economic aggressions perpetrated by children toward their parental figures (Pereira et al., 2017). Recent systematic reviews indicate that CPV can occur during childhood, adolescence, and even in young adulthood (Ibabe, 2020). In the present study, the term CPV is adopted to refer specifically to the dynamics between adolescents and their parental figures. Recently, there has been a noticeable increase in the number of families affected by CPV (Junco-Guerrero et al., 2025; Simmons et al., 2018), as well as a significant rise in the number of adolescents prosecuted for engaging in such violence, both in Spain and the United Kingdom (Fundación Amigó, 2025; Holt, 2022). A recent systematic review concluded that 34.8% of families experience CPV, with psychological aggression being the most prevalent form (Dahouri et al., 2025).

### 1.1. Cyber Child-to-Parent Violence: A New Form of Child-to-Parent Violence

The emergence of new communication tools, such as Information and Communication Technologies (ICTs), has facilitated the evolution of offline violence into new forms of violence occurring in online contexts. As a result, numerous scholars have focused on evaluating phenomena such as cyberbullying (Van Dam et al., 2012; Zych et al., 2015) and online dating violence (Caridade & Braga, 2020; Li et al., 2023). However, there remains a significant gap in the literature regarding cyber violence in family contexts (Rogers & Ashworth, 2024). Empirical studies of Cyber Child-to-Parent Violence (Cyber-CPV) are also scarce (Rodriguez-Gonzalez et al., n.d.-a; Rogers & Ashworth, 2024; Suárez-Relinque & Del Moral-Arroyo, 2023). Cyber-CPV refers to all forms of aggression perpetrated through ICTs by children, adolescents, or young adults toward their parental figures. These include direct online aggressions (e.g., online insults, threats, ignoring and/or blocking parental figures on digital platforms, and online control) as well as impersonation of parental figures in online settings. This type of violence is situated in the parent–child relationship, regardless of the child’s chronological age (Rodriguez-Gonzalez et al., n.d.-a). Recently, Cyber-CPV has been explored using a qualitative approach, with a focus on the perspectives of adolescents and parental figures involved in CPV situations, as well as professionals working in CPV interventions (Rodriguez-Gonzalez et al., n.d.-a).

Given the patterns observed in research on other forms of cyber violence, such as cyberbullying and online dating violence (Caridade & Braga, 2020; Machado et al., 2022), and the available data on Cyber-CPV (Rogers & Ashworth, 2024; Suárez-Relinque & Del Moral-Arroyo, 2023), it is possible to suggest that the latter phenomenon represents a new manifestation of CPV that shares developmental patterns with other cyber violence forms. In this regard, a bidirectional relationship has been observed between offline and online violence when referring to the same type of aggression (Borrajo & Gámez-Guadix, 2015; Khan et al., 2020). Moreover, CPV and Cyber-CPV appear to coexist in families (Rogers & Ashworth, 2024; Suárez-Relinque & Del Moral-Arroyo, 2023), which indicates a likely bidirectional association between the two.

However, cyber violence entails additional risks compared to its offline counterpart. First, cyber aggressions are often performed simultaneously through ICTs, which increases the frequency and reach of violent behaviors (Borrajo & Gámez-Guadix, 2015; Cava et al., 2020; Marganski & Melander, 2018; Stonard, 2020). Second, ICTs contribute to the emergence of more hostile and aggressive communication dynamics. This enables behaviors (e.g., insults and threats) that would be less likely to occur in face-to-face interactions. Also, these behaviors happen with greater intensity, aggression, and repetition (Lee et al., 2022; Schoffstall & Cohen, 2011). Third, ICTs eliminate spatial and temporal boundaries, thus facilitating constant contact; this can lead to prolonged aggression and controlling behaviors (Guidi et al., 2022).

Given the lack of knowledge regarding Cyber-CPV, it is necessary to draw on theoretical models and previous findings concerning traditional CPV (Bushman & Huesmann, 2010; Junco-Guerrero et al., 2025). This approach can guide the investigation of potential risk factors associated with this new form of CPV.

CPV has been studied in relation to a variety of individual (Calvete et al., 2020; Cuervo, 2025) and family-related (Cortina & Martín, 2020; Junco-Guerrero et al., 2025) risk factors. Regarding the former, some scholars have noted that internalizing symptoms—particularly depression and anxiety (Martínez-Ferrer et al., 2020)—are highly prevalent in CPV (Calvete et al., 2012; Martínez-Ferrer et al., 2020). Adolescents involved in CPV dynamics frequently exhibit depressive and anxious symptomatology (Armstrong et al., 2018; Biehal, 2012). In line with this, other authors have identified internalizing symptoms as strong predictors of CPV (Calvete et al., 2012; Jiménez-Granado et al., 2025). The adolescents in question also tend to have low self-esteem (Ibabe et al., 2014; Pereira & Bertino, 2009). Based on the existing evidence linking internalizing symptomatology and low self-esteem with CPV, it is reasonable to explore the potential bidirectionality between distress—depressive and anxious symptoms, low self-esteem, and reduced well-being (Pechorro et al., 2023)—and Cyber-CPV. Substance abuse has also emerged as a clear longitudinal predictor of CPV (see the meta-analysis by Dahouri et al., 2025). Furthermore, when substance abuse interacts with psychological distress in adolescents, it intensifies CPV (for a review, see Simmons et al., 2018).

Regarding family-related risk factors, exposure to family violence (Armstrong et al., 2021; Calvete et al., 2020; Cano-Lozano et al., 2024) and the use of punitive discipline—including physical and psychological punishment (Beckmann et al., 2021; Del Hoyo-Bilbao et al., 2018; Ibabe, 2019)—stand out as key contributors to the development of CPV. Importantly, it is not only the use of discipline that is relevant to understanding the development of this form of intrafamilial violence, but also the context in which punishment is applied and the manner in which it is enacted (Cano-Lozano et al., 2021; Del Hoyo-Bilbao et al., 2020). In this regard, parental impulsivity—defined as the tendency to respond in an uncontrolled and unreflective manner to children’s problematic behaviors—has been identified as a predictor of CPV (Cano-Lozano et al., 2021; Dahouri et al., 2025; Del Hoyo-Bilbao et al., 2020). Similarly, parental ineffectiveness, understood as the inability to establish clear rules and adequately manage inappropriate behaviors, has also been associated with a greater risk of CPV (Cano-Lozano et al., 2021; Del Hoyo-Bilbao et al., 2020). Therefore, it is reasonable to expect that these family-related factors may also play a role in the manifestation of Cyber-CPV.

### 1.2. The Present Study

CPV has been examined in relation to multiple individual (Armstrong et al., 2021; Beckmann et al., 2021) and family-related (Calvete et al., 2020; Del Hoyo-Bilbao et al., 2020) risk factors. However, to date, scholars have not explored new forms of this type of intrafamilial violence involving the use of ICTs, which has created a considerable gap in the literature on Cyber-CPV. Currently, the limited empirical evidence available on Cyber-CPV is predominantly descriptive and stems from qualitative studies (Rodriguez-Gonzalez et al., n.d.-a; Suárez-Relinque & Del Moral-Arroyo, 2023). Hence, there is a pressing need to better understand the development of Cyber-CPV by using quantitative methodologies and drawing on the existing evidence on traditional CPV.

This study had three main objectives. The first one was to examine potential bidirectional relationships between Cyber-CPV and CPV, as well as between Cyber-CPV and distress, in adolescents. Based on studies showing bidirectional associations between different forms of offline and online violence (Jauregizar et al., 2024; Khan et al., 2020) and those indicating the coexistence of CPV and Cyber-CPV (Suárez-Relinque & Del Moral-Arroyo, 2023), a bidirectional relationship between these forms of violence is expected. Likewise, internalizing symptoms and low self-esteem have shown both high prevalence and predictive value in relation to CPV; therefore, a bidirectional relationship between distress and Cyber-CPV is also anticipated. The second objective was to analyze individual risk factors (distress and substance abuse) and family-related risk factors (exposure to family violence, punitive discipline, parental ineffectiveness, and parental impulsivity) associated with Cyber-CPV. Given the close link between these factors and CPV (Junco-Guerrero et al., 2025; Rogers & Ashworth, 2024), it is expected that the former will also predict Cyber-CPV. The third objective was to examine the previously mentioned individual and family-related factors associated with CPV. It is expected that the findings will align with the previous literature, indicating that family-related factors exert a substantial influence on CPV (Cano-Lozano et al., 2021; Contreras & Cano, 2015), while individual factors significantly predict its occurrence (Beckmann et al., 2021; Calvete et al., 2020). Given prior findings on the moderating role of substance abuse in the relationship between distress and CPV (Simmons et al., 2018), the fourth objective was to assess whether substance abuse moderates the relationship between distress and Cyber-CPV, with the expectation that it will intensify this association. In addition to these objectives, the study also examined the prevalence of mild and severe Cyber-CPV in the general population of Spanish adolescents.

## 2. Materials and Methods

### 2.1. Participants

The sample for this study was collected in two waves, with a six-month interval between Wave 1 (W1) and Wave 2 (W2). The final sample, which consisted of adolescents who completed both waves, comprised 1034 individuals. Of these, 531 were boys (43.7%); 500 were girls (41.2%), and three reported other gender (0.2%). The adolescents were aged between 13 and 18 years (*M*_age_ = 15.05; *SD* = 1.53). The majority of them (90.90%) were from Spain. Attrition analysis indicated that compared to the adolescents who completed both waves (*n* = 1034), those who failed to complete W2 (*n* = 181; 14.90%) showed significantly higher mean scores in Cyber-CPV (*t*(223.248) = −2.38, *p* < 0.01), CPV (*t*(1212) = −1.58, *p* < 0.01), distress (*t*(222.230) = −2.54, *p* < 0.01), exposure to family violence (*t*(213.613) = −2.29, *p* < 0.01), parental ineffectiveness (*t*(214.870) = −2.57, *p* < 0.01), parental impulsiveness (*t*(1198) = −1.79, *p* < 0.01), punitive discipline (*t*(223.867) = −2.38, *p* < 0.01), and substance abuse (*t*(203.512) = −3.14, *p* < 0.01); they were also older (*t*(1213) = −4.04, *p* < 0.01). No statistically significant differences were found regarding gender, χ^2^(2) = 0.36, *p* = 0.84.

### 2.2. Procedure and Ethical Aspects

Educational institutions were contacted via phone and email, and they were informed about the nature of the study as well as its ethical and legal guarantees, including confidentiality, privacy, anonymity, and voluntary participation. The sample was obtained through collaboration agreements with educational authorities, ensuring a balanced representation of educational centers from both the public and semi-private sectors. The high schools were selected to reflect the diversity of the educational provision, and not through convenience sampling. Although the selection was not random in a strictly probabilistic sense, the process aimed to avoid sampling bias by including institutions from different geographic areas of the Basque Autonomous Community (BAC) and maintaining proportional representation of school types. No explicit stratification by socioeconomic level or urban/rural location was applied, but the final sample included centers from both urban and semi-urban areas. Eight schools (50% public and 50% private) agreed to participate. Additionally, 14 schools declined to participate in the study. Prior to data collection, parental figures were provided with a passive informed consent form, as minors’ participation did not involve any significant risk. Adolescents, as the primary participants, provided active consent in accordance with the principle of progressive autonomy and were informed that they could withdraw from the study at any time without explanation or consequence. This procedure safeguarded parental awareness while ensuring adolescents’ voluntary participation, thereby achieving an appropriate balance between the protection of minors and the feasibility of research in school settings. The study was reviewed and approved by the Ethics Committee of the University of Deusto [ETK-12/23-24], which confirmed the ethical suitability of the procedures and the absence of significant risks for participants. All students were invited to participate in the study; however, participation was voluntary and based on voluntary sign-up. A total of 27 adolescents declined to participate at W1, and 30 did so at W2. The questionnaires were administered during school hours using the Qualtrics software; completing them required approximately 50 min. Data collection sessions were supervised by trained research psychologists to provide support and clarification if needed. Furthermore, a contact number was made available to the adolescents and parents in case they had any queries regarding the questionnaires or any other issue connected to the study. To match the questionnaires from W1 and W2, a unique code known only to the participant was used. As a form of compensation, a 15-euro class-based raffle was held at each assessment wave.

### 2.3. Materials

#### 2.3.1. Cyber Child-to-Parent Violence

Cyber CPV was assessed using the version for adolescents of the Cyber Child-to-Parent Violence Questionnaire (Cyber-CPVQ-A; Rodriguez-Gonzalez et al., n.d.-b). This self-report instrument evaluates Cyber-CPV behaviors directed toward the mother and the father separately over the previous six months (e.g., “I have insulted her/him through message, audio, and/or call during an argument and/or when I was angry”); it uses 14 items with four response options that range from 0 (never) to 3 (always or occurred six times or more). At W1, the participants reported behaviors from the previous 12 months, while at W2, the reporting period was limited to the past six months. In the original validation, the scale demonstrated good psychometric properties, with a Cronbach’s alpha value of 0.85 (Rodriguez-Gonzalez et al., n.d.-b). In the present study, Cronbach’s alpha values were 0.82 at W1 and 0.85 at W2.

#### 2.3.2. Child-to-Parent Violence

CPV was assessed using the Child-to-Parent Aggression Questionnaire–Revised Version (CPAQ-R; Calvete et al., 2023). This self-report questionnaire consists of 18 items that evaluate CPV behaviors directed toward the mother and the father separately (e.g., “You have threatened to hit her/him, although you didn’t actually do it”). At W1, the participants reported on behaviors occurring over the previous 12 months, whereas at W2, the reference period was the past six months. Responses were rated on a four-point scale ranging from 0 (never) to 3 (always or occurred six times or more). The scale demonstrated good internal consistency; the Cronbach’s alpha values were 0.84 at W1 and 0.90 at W2, which are similar to those reported in studies with comparable samples (Jiménez-Granado et al., 2023a).

#### 2.3.3. Distress

Distress was assessed using the Weinberger Adjustment Inventory–Short Form (WAI-SF; Pechorro et al., 2023). This self-report questionnaire evaluates emotional adjustment at the present moment through 24 items rated on a five-point scale ranging from 1 (false/almost never) to 5 (true/almost always). The instrument includes two main scales: distress (12 items), which comprises the subscales for depression, anxiety, low well-being, and low self-esteem, and restraint (12 items), which includes the subscales for suppression of aggression, impulse control, responsibility, and consideration for others. In the present study, only the overall score of the distress scale was used. The original validation showed adequate internal consistency, with Cronbach’s alpha values exceeding the recommended threshold of 0.70 (Pechorro et al., 2023). In the present study, the alpha coefficients were 0.70 at W1 and 0.74 at W2.

#### 2.3.4. Exposure to Family Violence

Exposure to family violence was assessed using the Exposure to Violence Questionnaire (CEV; Orue & Calvete, 2010). This scale consists of six items that measure exposure to violence from the victim’s perspective (three items, e.g., “Someone has hit or physically harmed you at home”) and the witness’s perspective (three items, e.g., “You have seen someone hitting or physically harming another person at home”) in different settings (e.g., people’s homes, the streets, schools, and TV content). In this study, only the context referring to home environments was used. Responses are rated on a four-point scale ranging from 0 (never) to 5 (every day). The original validation reported a Cronbach’s alpha value of 0.86 (Orue & Calvete, 2010). In the present study, the scale demonstrated excellent psychometric properties, with Cronbach’s alpha value of 0.89 at W1.

#### 2.3.5. Punitive Discipline

Punitive discipline was assessed using the Spanish version of Section C of the Dimensions of Discipline Inventory (DDI-A; Straus & Fauchier, 2007; Spanish version by Calvete et al., 2010). This section includes 26 disciplinary strategies grouped into nine different dimensions. For the purposes of this study, only the punitive discipline subscale was used (e.g., “My mother/father shook me or grabbed me forcefully to make me obey” and “My mother/father yelled at me”). Responses were rated on a five-point scale ranging from 0 (never) to 5 (always or almost always), with participants responding based on their experiences at the age of 10. In this study, the punitive discipline subscale demonstrated excellent psychometric properties, with Cronbach’s alpha value of 0.91 at W1. This result aligns with the original validation, which reported an alpha coefficient of 0.81 (Calvete et al., 2010).

#### 2.3.6. Parental Ineffectiveness and Parental Impulsivity

Parental ineffectiveness and parental impulsivity were assessed using Section D of the Dimensions of Discipline Inventory (DDI-A; Straus & Fauchier, 2007). This section includes two primary scales: (1) the disciplinary context, comprising four subscales, and (2) the mode of implementation of disciplinary behavior, which includes six subscales. For the present study, only the subscales for parental ineffectiveness, as part of the discipline’s implementation context (three items, e.g., “My mother/father had trouble controlling my misbehavior”), and parental impulsivity, as part of the mode of discipline assessed (three items, e.g., “My mother/father felt like they lost control when I misbehaved”), were used. Responses were rated on a five-point scale ranging from 0 (never) to 5 (always or almost always), with participants responding based on their experiences at the age of 10. In the present study, the internal consistency was excellent, with Cronbach’s alpha values of 0.86 for parental ineffectiveness and 0.80 for parental impulsivity. In previous studies with comparable samples, similar reliability estimates were reported. For instance, Del Hoyo-Bilbao et al. (2020) found Cronbach’s alpha values of 0.88 for the parental ineffectiveness subscale and 0.76 for the parental impulsivity subscale.

#### 2.3.7. Substance Abuse

Substance abuse was assessed using the Adolescent Drug Abuse Inventory (ADAI; Calvete & Estévez, 2009). The participants reported how frequently they used eight types of substances (e.g., tobacco, alcohol, marijuana, hashish, ecstasy, cocaine, speed, and ketamine). Their responses were recorded on a seven-point scale ranging from 0 (never) to 7 (daily). The ADAI demonstrated good psychometric properties, with a Cronbach’s alpha value of 0.82. The scale also demonstrated adequate internal consistency, with a Cronbach’s alpha of 0.80, which is in line with previous research involving adolescent samples (Calvete et al., 2020).

### 2.4. Data Analysis

**Figure 1:**
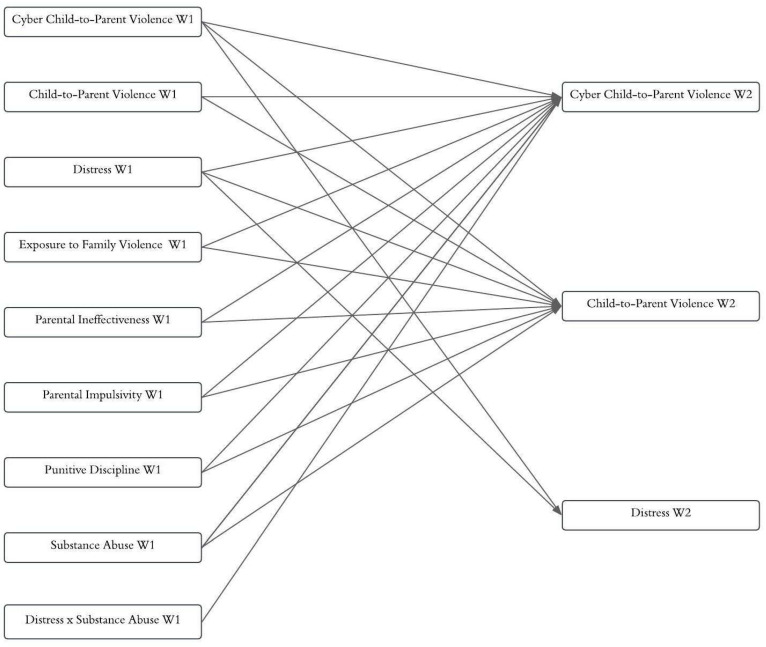
Hypothetical Model. Note. Although all covariances were estimated in the model, only those related to the outcome variables are presented in the figure for the sake of clarity and ease of interpretation.

First, IBM SPSS Statistics (version 28.0) was used to perform descriptive analyses and Spearman correlations among the study variables. Then, LISREL (version 8.80) was employed to perform the path analysis for evaluating the hypothetical model. Data were analyzed with the robust maximum likelihood estimator, and the Satorra–Bentler scaled chi-square (S-B χ^2^) method was applied (Jöreskog & Sörbom, 2006). The hypothetical model (Figure 1) included measures of Cyber-CPV, CPV, and distress at W1 and W2, focusing specifically on the bidirectional associations between CPV and Cyber CPV, as well as between distress and Cyber CPV. The model included autoregressive paths from W1 to W2 to control for the effects of the variable over time. In addition, the predictive effects of exposure to family violence, parental ineffectiveness, parental impulsivity, punitive discipline, and substance abuse at W1 on both Cyber-CPV and CPV at W2 were examined. An interaction term was also created to assess the moderating effect of distress and substance abuse (distress × substance abuse) at W1, following established methodologies for generating moderation terms. For this purpose, the distress and substance abuse variables were transformed into z-scores. Finally, differences between the estimated slopes were analyzed using Dawson’s (2018) approach. Goodness of fit was assessed based on the criteria recommended by Little (2013), where NNFI values of 0.90 or higher, RMSEA values of 0.06 or lower, CFI values of 0.90 or higher, and SRMR values below 0.08 are considered indicative of good model fit.

## 3. Results

### 3.1. Descriptive Analysis

Table 1 presents the results of the correlation analyses, along with the means and standard deviations. The Kolmogorov–Smirnov test (Massey, 1951) indicated that the data did not follow a normal distribution for the variables Cyber-CPV, distress, and punitive discipline. Therefore, Spearman’s rank-order correlation was used to estimate the associations. All the study variables showed significant and positive correlations. The prevalence of general Cyber-CPV at W1 was 78.7%, and severe Cyber-CPV was reported by 22% of participants. At W2, the prevalence of general Cyber-CPV was 76.1%, while the prevalence of severe Cyber-CPV was 25.9%. To assess severe violence, adolescents who reported having engaged in violent behavior six times or more in the past year were considered.

### 3.2. Predictive Model

**Figure 2:**
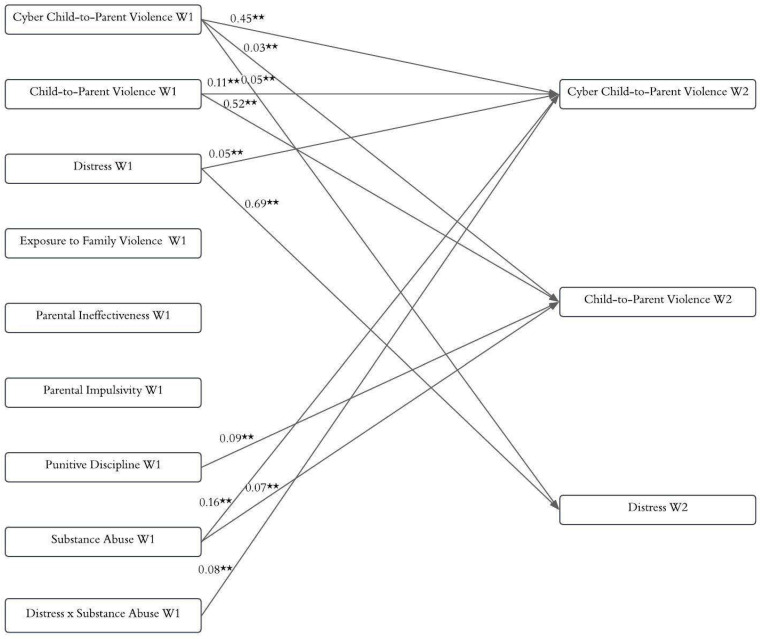
Parsimonious Model. Note. Although all covariances were estimated in the model, only those related to the outcome variables are presented in the figure for the sake of clarity and ease of interpretation. ** *p* < 0.001.

**Figure 3:**
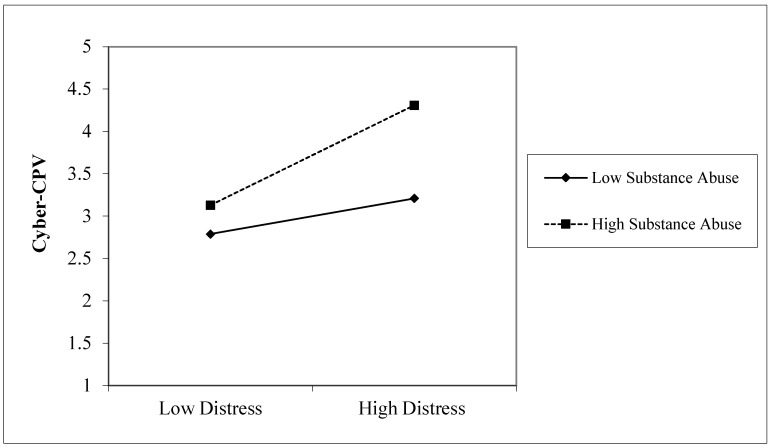
Two Wave Interaction of Substance Abuse and Distress across Cyber-CPV. Note: Cyber Child-to-Parent Violence (Cyber-CPV).

Figure 1 shows the hypothetical model in which bidirectional relationships between Cyber-CPV, CPV, and distress were analyzed. Cross-lagged paths were estimated, including reciprocal effects between the variables assessed at W1 and W2. The model also included autoregressive paths for Cyber-CPV, CPV, and distress, as well as cross-lagged paths for the family-related variables (exposure to family violence, parental ineffectiveness, parental impulsivity, and punitive discipline) and the individual variable substance abuse, all measured at W1, as predictors of Cyber-CPV and CPV at W2. This allowed the identification of the factors associated with changes in Cyber-CPV over time. The hypothesized model yielded the following fit indices: S-B χ^2^(22, *n* = 1034) = 24.37, RMSEA = 0.01 (90% CI [0.00, 18.67]), NNFI = 1.00, CFI = 1.00, and SRMR = 0.03. Then, the nonsignificant paths were eliminated to create a more parsimonious model. The final model (see Figure 2) showed excellent fit indices: S-B χ^2^(16, *n* = 1034) = 14.78, RMSEA = 0.0 (90% CI [0.00, 11.80]), NNFI = 1.00, CFI = 1.00, and SRMR = 0.03. The model explained 37.00% of the variance in Cyber-CPV, 36.00% in CPV, and 49.00% in distress. All the autoregressive paths were statistically significant, indicating the stability of the variables over the six-month interval. Figure 2 illustrates two bidirectional relationships: (1) Cyber-CPV at W1 did not predict CPV at W2, whereas CPV at W1 did predict Cyber-CPV at W2. (2) Cyber-CPV at W1 predicted distress at W2, while distress at W1 predicted Cyber-CPV at W2. Substance abuse at W1 significantly predicted both Cyber-CPV and CPV at W2, whereas punitive discipline at W1 predicted only CPV at W2. Additionally, the interaction term (distress × substance abuse) at W1 predicted Cyber-CPV at W2. As shown in Figure 3, when both distress and substance abuse were high, levels of Cyber-CPV increased significantly (Slope 1 [high substance abuse]: β = 0.59, *t* = 3.11, *p* < 0.01). In contrast, when substance abuse was low, distress did not significantly predict Cyber-CPV (Slope 2 [low substance abuse]: β = 0.21, *t* = 1.57, *p* = 0.12). Exposure to family violence, parental impulsivity, and punitive discipline at W1 were not significantly associated with Cyber-CPV at W2.

## 4. Discussion

The present study had four main objectives. The first one was to examine bidirectional relationships between Cyber-CPV and CPV, as well as between Cyber-CPV and distress. The second objective was to assess individual risk factors (distress and substance abuse) and family-related risk factors (exposure to family violence, parental ineffectiveness, parental impulsivity, and punitive discipline) in relation to Cyber-CPV. The third objective focused on examining the previously mentioned individual and family-related factors associated with CPV. The fourth objective was to evaluate the moderating role of substance abuse in the relationship between distress and Cyber-CPV. The results did not provide evidence of a bidirectional relationship between Cyber-CPV and CPV; however, CPV showed significant predictive effects on Cyber-CPV. Additionally, the findings reveal a bidirectional association between Cyber-CPV and distress. Furthermore, substance abuse significantly predicted Cyber-CPV over time and intensified the relationship between distress and Cyber-CPV. Additionally, both punitive discipline and substance abuse showed significant predictive relationships with CPV.

First, although a bidirectional relationship between Cyber-CPV and CPV was not found, CPV showed a significant predictive effect on Cyber-CPV. This finding aligns with previous research showing that offline violence is a strong predictor of its online counterpart in online dating violence (Borrajo & Gámez-Guadix, 2015; Schokkenbroek et al., 2022) and cyberbullying (Camacho et al., 2023). In this regard, consistent with findings on other forms of cyber violence, violent behaviors perpetrated offline may transfer to online contexts, where the absence of face-to-face interaction can both aggravate the harm inflicted on parental figures and facilitate the expression of aggression. (Lee et al., 2022; Stonard, 2020). These findings align with cyberbullying research showing that the absence of spatial and temporal barriers can intensify online aggression and reduce adolescents’ awareness of its impact (Guidi et al., 2022; Schoffstall & Cohen, 2011). Furthermore, the lack of a reverse predictive relationship—where online aggression predicts CPV—may indicate that aggression is more easily expressed in digital contexts, whereas face-to-face interactions involve additional emotional, social, and situational components that can act as inhibitors (Muñoz-Fernández & Sánchez-Jiménez, 2020). Thus, Cyber-CPV can be conceptualized as a distinct form of violence, characterized by specific features and primarily shaped by individual factors, whereas offline CPV is more strongly associated with family dynamics, particularly parenting practices and punitive discipline.

Second, and in line with expectations, bidirectional relationships were found between Cyber-CPV and distress. This result is consistent with previous research indicating that internalizing symptomatology—particularly depressive and anxious symptoms—contributes to greater perpetration of CPV (Calvete et al., 2012; Jiménez-Granado et al., 2025). However, in a recent study, no significant bidirectional associations between internalizing symptoms and CPV were found (Jiménez-Granado et al., 2025). This discrepancy may be due to the fact that the authors of such a study focused only on depressive and anxious symptoms, overlooking other relevant dimensions of distress, such as low self-esteem and reduced well-being, both of which were included in the present study. The findings of the latter suggest that low self-esteem and diminished well-being play a meaningful role in the bidirectional relationship between distress and Cyber-CPV, which highlights the importance of a broader conceptualization of distress in future research.

Third, and consistent with our hypothesis, this study revealed predictive relationships between substance abuse and both Cyber-CPV and CPV (Calvete et al., 2020). This result aligns with previous research on CPV, where substance abuse has been identified as a strong predictor of CPV (Calvete et al., 2020; Ruiz-Fernández et al., 2021). Moreover, this study found that levels of Cyber-CPV were significantly higher in contexts of high distress and high substance abuse, compared to situations characterized by low levels of both. This finding is in line with systematic reviews highlighting the facilitating role of substance abuse in the escalation of CPV, particularly among adolescents with internalizing symptomatology (Junco-Guerrero et al., 2025; Simmons et al., 2018). One possible explanation for this evidence is that adolescents experiencing higher levels of emotional distress—including depressive and anxious symptoms—may be more likely to engage in substance abuse as a dysfunctional coping strategy (Park & Kim, 2016; Tian et al., 2021). This may contribute to the development of aggressive behaviors. Overall, the results of the present study show that substance abuse is a key factor in the development of Cyber-CPV, as it acts as an aggravating element in contexts of elevated adolescent distress. Hence, substance abuse is of high relevance in understanding this form of violence.

Fourth, and contrary to expectations, no predictive relationships were found between the family-related variables—exposure to family violence, parental ineffectiveness, parental impulsivity, and punitive discipline—and Cyber-CPV, notably, punitive discipline emerged as the only family-related factor exhibiting predictive associations with CPV (Del Hoyo-Bilbao et al., 2020), which is consistent with the findings reported in the previous literature. This suggests that while CPV appears to be closely associated with family and contextual factors (Beckmann et al., 2021), Cyber-CPV may be more strongly linked to adolescents’ individual characteristics. It is worth noting that the family environment plays a crucial role in CPV, as face-to-face interactions with parental figures may reflect socialization patterns and family dynamics (Calvete et al., 2011; Contreras & Cano-Lozano, 2014). This may explain why exposure to family violence (Calvete et al., 2020; Izaguirre & Calvete, 2017) and punitive discipline (Cano-Lozano et al., 2021; Del Hoyo-Bilbao et al., 2020) have been identified as strong predictors of CPV. In contrast, Cyber-CPV is enacted through ICTs, where direct contact with the target at the moment of aggression is diminished; this potentially grants the perpetrator a greater sense of emotional distance (Guidi et al., 2022). In this context, individual factors, such as distress or substance abuse, seem to play a more prominent role. Thus, although the family environment remains relevant, its weight in relation to Cyber-CPV appears to be less significant compared to what is the case in traditional CPV. This may be due to the fact that Cyber-CPV does not require face-to-face confrontation and allows for a more disinhibited expression of violence. Another aim of this study was to provide data on the prevalence of Cyber-CPV among adolescents in Spain. Between 76.1% and 78.7% of the participants reported having engaged in some form of Cyber-CPV, and between 22% and 25.9% reported engaging in such behavior six times or more over the previous six months. These figures are slightly higher than those typically reported for CPV in adolescent populations, particularly when compared to psychological CPV, with which Cyber-CPV shares similar patterns of manifestation (Calvete et al., 2023; Dahouri et al., 2025). This may be explained by the characteristics of ICTs, such as the absence of spatial and temporal boundaries associated with their use, which facilitate the perpetration of aggression (Guidi et al., 2022; Jauregizar et al., 2024). In any case, the findings of the present study highlight Cyber-CPV as an emerging issue among adolescents, which warrants further empirical investigation to identify its associated risk factors and better understand the phenomenon.

## 5. Limitations and Future Directions

This study has several limitations that should be acknowledged. First, the data came entirely from self-reports. Given that Cyber-CPV is a family issue involving multiple agents, future researchers should incorporate parental reports to provide a more comprehensive understanding of the phenomenon (Jiménez-Granado et al., 2023b; Calvete et al., 2017). Second, the variables were assessed at two time points, with a six-month interval between the measurements. Future scholars should replicate this design using a longer time span, which may allow the detection of associations with family-related variables, as these may require more time to become empirically observable. Third, although this study was focused on risk factors for Cyber-CPV, there is a clear need for more data on protective factors. Research on this aspect is still in its infancy, and scholars’ empirical understanding of it remains limited. This makes it difficult to adequately assess protective elements. Therefore, future researchers should explore this aspect to provide a more holistic perspective on Cyber-CPV and inform the development of effective prevention and intervention strategies. Fourth, this study relied on only two time points, which restricts the ability to capture more complex patterns of change over time. Future research should incorporate at least three waves to more accurately examine developmental trajectories and to allow for the application of more sophisticated longitudinal analyses, such as robust cross-lagged panel models. Fifth, no formal stratification was carried out in the sampling process, which may affect the generalizability of the results. Future research should consider implementing specific stratification procedures that take into account variables such as geographical location, socioeconomic status, and school type, thereby ensuring the representativeness of the sample with respect to these factors. Sixth, although the instruments employed have been validated in similar populations, future research should include Confirmatory Factor Analysis (CFA) to further examine their factorial structure and provide additional evidence regarding the validity of the measurement. In light of the methodological limitations of this study, the findings should be considered preliminary and interpreted with caution.

## 6. Strengths and Practical Implications

This study has several strengths. First, it provides the first longitudinal evidence on Cyber-CPV, as well as initial data on its prevalence among adolescents. As such, it represents an innovative contribution to the empirical and theorical literature on CPV research. In addition, the study offers evidence of bidirectional relationships between Cyber-CPV and CPV, which suggests that Cyber-CPV may be part of the developmental trajectory of CPV and could be considered a new manifestation of this form of intrafamilial violence (Suárez-Relinque & Del Moral-Arroyo, 2023). The bidirectional associations between Cyber-CPV and distress indicate that adolescent distress may trigger aggressive behaviors, which may reinforce emotional distress. This highlights the importance of addressing adolescent distress, as it may contribute to a cyclical pattern that perpetuates Cyber-CPV. Furthermore, substance abuse was found to intensify Cyber-CPV in situations of high distress, indicating the need to pay particular attention to adolescent substance abuse—especially in contexts of emotional discomfort—since it may serve as a maladaptive coping strategy that contributes to violent online behaviors toward parental figures.

These findings can inform the design of prevention and adolescent health promotion programs, as Cyber-CPV behaviors may act as precursors of CPV. From a psychoeducational perspective, the results of this study may be useful for raising awareness among adolescents, families, therapists, and educators about the existence and implications of Cyber-CPV. Additionally, the findings underscore the importance of promoting healthy and responsible ICT use, which should be integrated into preventive programs addressing online aggression and encouraging positive digital behaviors.

## Figures and Tables

**Table 1:** Spearman Correlations Among the Study Variables.

	1	2	3	4	5	6	7	8	9	10	11
1. Cyber Child-to-Parent Violence W1	1										
2. Cyber Child-to-Parent Violence W2	0.58 **	1									
3. Child-to-Parent Violence W1	0.48 **	36 **	1								
4. Child-to-Parent Violence W2	0.37 **	49 **	0.59 **	1							
5. Distress W1	0.24 **	0.19 **	0.25 **	0.24 **	1						
6. Distress W2	0.22 **	0.28 **	0.17 **	0.24 **	0.70 **	1					
7. Exposure to Family Violence W1	0.37 **	0.28 **	0.46 **	0.38 **	0.35 **	0.31 **	1				
8. Parental Ineffectiveness W1	0.32 **	0.24 **	0.34 **	0.29 **	0.25 **	0.18 **	0.31 **	1			
9. Parental Impulsiveness W1	0.21 **	0.14 **	0.22 **	0.20 **	0.14 **	0.10 *	0.32 **	0.36 **	1		
10. Punitive Discipline W1	0.48 **	0.32 **	0.43 **	0.37 **	0.37 **	0.28 **	0.63 **	0.46 **	0.39 **	1	
11. Substance Abuse W1	0.29 **	0.24 **	0.18 **	0.16 **	0.17 **	0.16 **	0.21 **	0.14 *	0.10 *	0.24 **	1

*Note.* * *p* < 0.05; ** *p* < 0.001.

## Data Availability

The data supporting the findings of this study are available from the corresponding author upon request, due to ethical considerations and restrictions related to participant confidentiality.

## References

[B1-ejihpe-15-00190] Armstrong G. S., Cain C. M., Wylie L. E., Muftić L. R., Bouffard L. A. (2018). Risk factor profile of youth incarcerated for child to parent violence: A nationally representative sample. Journal of Criminal Justice.

[B2-ejihpe-15-00190] Armstrong G. S., Muftić L. R., Bouffard L. A. (2021). Factors influencing law enforcement responses to child to parent violence. Journal of Interpersonal Violence.

[B3-ejihpe-15-00190] Beckmann L., Bergamann M. C., Fischer F., Mößle T. (2021). Risk and protective factors of child-to-parent violence: A comparison between physical and verbal aggression. Journal of Interpersonal Violence.

[B4-ejihpe-15-00190] Biehal N. (2012). Parent abuse by young people on the edge of care: A child welfare perspective. Social Policy and Society.

[B5-ejihpe-15-00190] Borrajo E., Gámez-Guadix M. (2015). Cyber dating abuse: Prevalence, context, and relationship with offline dating aggression. Psychological Reports: Relationships & Communications.

[B6-ejihpe-15-00190] Bushman B. J., Huesmann L. R., Fiske S. T., Gilbert D. T., Lindzey G. (2010). Aggression. Handbook of social psychology.

[B7-ejihpe-15-00190] Calvete E., Estévez A. (2009). Substance abuse in adolescents: The role of stress, impulsivity, and schemas related to lack of boundaries. Addictions.

[B8-ejihpe-15-00190] Calvete E., Gámez-Guadix M., Orue I. (2010). The dimensions of discipline inventory (DDI)-child and adolescent version: Analysis of the parental discipline from a gender perspective. Anales De Psicología.

[B9-ejihpe-15-00190] Calvete E., Jiménez-Granado A., Orue I. (2023). The revised child-to-parent aggressions questionnaire: An examination during the COVID-19 pandemic. Journal of Family Violence.

[B10-ejihpe-15-00190] Calvete E., Orue I., Fernández-González L., Chang R., Little T. D. (2020). Longitudinal trajectories of child-to-parent violence through adolescence. Journal of Family Violence.

[B11-ejihpe-15-00190] Calvete E., Orue I., Gámez-Guadix M. (2012). Child-to-parent violence: Emotional and behavioral predictors. Journal of Interpersonal Violence.

[B12-ejihpe-15-00190] Calvete E., Orue I., González-Cabrera J. (2017). Child-to-parent violence: Comparing reports from adolescents and their parents. Journal of Clinical Psychology with Children and Adolescents.

[B13-ejihpe-15-00190] Calvete E., Orue I., Sampedro R. (2011). Child-to-parent violence in adolescence: Environmental and personal characteristics. Childhood and Learning.

[B14-ejihpe-15-00190] Camacho A., Runions K., Ortega-Ruiz R., Romera E. M. (2023). Bullying and cyberbullying perpetration and victimization: Prospective with-person associations. Journal of Youth and Adolescence.

[B15-ejihpe-15-00190] Cano-Lozano M. C., León S. P., Contreras L. (2021). Relationship between punitive discipline and child-to-parent violence: The moderating role of the context and implementation of parenting practices. International Journal of Environmental Research and Public Health.

[B16-ejihpe-15-00190] Cano-Lozano M. C., Navas-Martínez M. J., Contreras L. (2024). Lagged and simultaneous effects of exposure to violence at home on child-to-parent violence: Gender differences. Frontiers in Psychiatry.

[B17-ejihpe-15-00190] Caridade S. M. M., Braga T. (2020). Youth cyber dating abuse: A meta-analysis of risk and protective factors. Cyberpsychology: Journal of Psychosocial Research on Cyberspace.

[B18-ejihpe-15-00190] Cava M. J., Buelga S., Carrascosa L., Ortega-Barón J. (2020). Relations among romantic myths, offline dating violence victimization and cyber dating violence victimization in adolescents. International Journal of Environmental Research and Public Health.

[B19-ejihpe-15-00190] Contreras L., Cano M. C. (2015). Exploring psychological features in adolescents who assault their parents: A different profile of young offenders?. The Journal of Forensic Psychiatry & Psychology.

[B20-ejihpe-15-00190] Contreras L., Cano-Lozano M. C. (2014). Family profile of young offenders who abuse their parents: A comparison with general offenders and non-offenders. Journal of Family Violence.

[B21-ejihpe-15-00190] Cortina H., Martín A. M. (2020). The behavioral specificity of child-to-parent violence. Annals of Psychology.

[B22-ejihpe-15-00190] Cuervo K. (2025). Risk factor profile in child-to-parent violence: A gender analysis. Criminal Justice and Behavior.

[B23-ejihpe-15-00190] Dahouri A., Mirghafourvand M., Zahedi H., Maghalian M., Hosseinzadeh M. (2025). Prevalence of child to parent violence and its determinants: A systematic review and meta-analysis. BMC Public Health.

[B24-ejihpe-15-00190] Dawson J. F. (2018). Interpreting interaction effects.

[B25-ejihpe-15-00190] Del Hoyo-Bilbao J., Gámez-Guadix M., Calvete E. (2018). Corporal punishment by parents and child-to-parent aggression in Spanish adolescents. Annals of Psychology.

[B26-ejihpe-15-00190] Del Hoyo-Bilbao J., Orue I., Gámez-Guadix M., Calvete E. (2020). Multivariate models of child-to-mother violence and child-to-father violence among adolescents. The European Journal of Psychology Applied to Legal Context.

[B27-ejihpe-15-00190] Fundación Amigó (2025). Child-to-parent violence in Spain.

[B28-ejihpe-15-00190] Guidi S., Palmitesta P., Bracci M., Marchigiani E., Di Pomponio I., Parlangeli O. (2022). How many cyberbullying(s)? A non-unitary perspective for offensive online behaviours. PLoS ONE.

[B29-ejihpe-15-00190] Holt A. (2022). Child to parent abuse.

[B30-ejihpe-15-00190] Ibabe I. (2019). Adolescent-to-parent violence and family environment: The perceptions of same reality?. International Journal of Environmental Research and Public Health.

[B31-ejihpe-15-00190] Ibabe I. (2020). A systematic review of youth-to-parent aggression: Conceptualization, typologies, and instruments. Frontiers in Psychology.

[B32-ejihpe-15-00190] Ibabe I., Arnoso A., Elgorriaga E. (2014). Behavioral problems and depressive symptomatology as predictors of child-to-parent violence. European Journal of Psychology Applied to Legal Context.

[B33-ejihpe-15-00190] Izaguirre A., Calvete E. (2017). Exposure to family violence as a predictor of dating violence and child-to-parent aggression in Spanish adolescents. Youth & Society.

[B34-ejihpe-15-00190] Jauregizar J., Dosil-Santamaria M., Redondo I., Wasch S., Machimbarrena J. M. (2024). Online and offline dating violence: Same same, but different?. Psicologia: Reexão E Crítica.

[B35-ejihpe-15-00190] Jiménez-Granado A., del Hoyo-Bilbao J., Fernández-González L. (2023a). Interaction of parental discipline strategies and adolescents’ personality traits in the prediction of child-to-parent violence. European Journal of Psychology Applied to Legal Context.

[B36-ejihpe-15-00190] Jiménez-Granado A., Fernández-González L., del Hoyo-Bilbao J. (2025). Longitudinal reciprocal associations between internalizing symptoms and child-to-parent violence in adolescents: The role of cognitive mechanisms. Psychology of Violence.

[B37-ejihpe-15-00190] Jiménez-Granado A., Fernández-González L., del Hoyo-Bilbao J., Calvete E. (2023b). Psychological symptoms in parents who experience child-to-parent violence: The role of self-efficacy beliefs. Healthcare.

[B38-ejihpe-15-00190] Jöreskog K. G., Sörbom D. (2006). LISREL 8.80 for windows *[Computer software]*.

[B39-ejihpe-15-00190] Junco-Guerrero M., Fernández-Baena F. J., Cantón-Cortés D. (2025). Risk factors for child-to-parent violence: A scoping review. Journal of Family Violence.

[B40-ejihpe-15-00190] Khan F., Limbana T., Zahid T., Eskander N., Jahan N. (2020). Traits, trends, and trajectory of tween and teen cyberbullies. Curēus.

[B41-ejihpe-15-00190] Lee Y., Harris M., Kim J. (2022). Gender differences in cyberbullying victimization from a developmental perspective: An examination of risk and protective factors. Crime and Delinquency.

[B42-ejihpe-15-00190] Li J., Ran G., Zhang Q., He X. (2023). The prevalence of cyber dating abuse among adolescents and emerging adults: A meta-analysis. Computers in Human Behavior.

[B43-ejihpe-15-00190] Little T. D. (2013). Longitudinal structural equation modeling.

[B44-ejihpe-15-00190] Machado B., Caridade S., Araújo I., Lobato-Faria P. (2022). Mapping the cyber interpersonal violence among young populations: A scoping review. Social Sciences.

[B45-ejihpe-15-00190] Marganski A., Melander L. (2018). Intimate partner violence victimization in the cyber and real world: Examining the extent of cyber aggression experiences and its association with in-person dating violence. Journal of Interpersonal Violence.

[B46-ejihpe-15-00190] Martínez-Ferrer B., Romero-Abrio A., León-Moreno C., Villarreal-González M. E., Musitu-Ferrer D. (2020). Suicidal ideation, psychological distress and child-to-parent violence: A gender analysis. Frontiers in Psychology.

[B47-ejihpe-15-00190] Massey F. J. (1951). The Kolmogorov-Smirnov test for goodness of fit. Journal of the American Statistical Association.

[B48-ejihpe-15-00190] Muñoz-Fernández N., Sánchez-Jiménez V. (2020). Cyber-aggression and psychological aggression in adolescent couples: A short-term longitudinal study on prevalence and common and differential predictors. Computers in Human Behavior.

[B49-ejihpe-15-00190] Orue I., Calvete E. (2010). Development and validation of a questionnaire to measure exposure to violence in childhood and adolescence. International Journal of Psychology and Psychological Therapy.

[B50-ejihpe-15-00190] Park S., Kim Y. (2016). Prevalence, correlates, and associated psychological problems of substance use in Korean adolescents. BMC Public Health.

[B51-ejihpe-15-00190] Pechorro P., DeLisi M., Freitas A., Abrunhosa Gonzçalvez R., Nunes C. (2023). Examination of the Weinberger adjustment inventory–short form among Portuguese young adults: Psychometrics and measurement invariance. International Journal of Offender Therapy and Comparative Criminology.

[B52-ejihpe-15-00190] Pereira R., Bertino L. (2009). An ecological understanding of child-to-parent violence. Journal of Relational Psychotherapy and Social Interventions.

[B53-ejihpe-15-00190] Pereira R., Loinaz I., Del Hoyo-Bilbao J., Arospide J., Bertino L., Calvo A., Montes Y., Guiterrez M. M. (2017). Proposal for a definition of filio-parental violence: Consensus of the Spanish society for the study of filio-parental violence (SEVIFIP). Papeles Del Psicólogo.

[B54-ejihpe-15-00190] Rodriguez-Gonzalez S., Del Hoyo-Bilbao J., Echezarraga A. (n.d.-a). Cyber child-to-parent violence: A qualitative study from the perspective of adolescents, parental figures and professionals.

[B55-ejihpe-15-00190] Rodriguez-Gonzalez S., Del Hoyo-Bilbao J., Echezarraga A., Fernández-González L. (n.d.-b). Cyber child-to-parent violence: Assessment and prevalence according to adolescents’ and parent’s reports.

[B56-ejihpe-15-00190] Rogers M. M., Ashworth C. (2024). Child-to-parent violence and abuse: A scoping review. Trauma, Violence, & Abuse.

[B57-ejihpe-15-00190] Ruiz-Fernández A., Junco-Guerrero M., Cantón-Cortés D. (2021). Exploring the mediating effect of psychological engagement on the relationship between child-to-parent violence and violent video games. International Journal of Environmental Research and Public Health.

[B58-ejihpe-15-00190] Schoffstall C. L., Cohen R. (2011). Cyber aggression: The relation between online offenders and offline social competence. Social Development.

[B59-ejihpe-15-00190] Schokkenbroek J. M., Van Ouytsel J., Hardyns W., Ponnet K. (2022). Adults’ online and offline psychological intimate partner violence experiences. Journal of Interpersonal Violence.

[B60-ejihpe-15-00190] Simmons M., McEwan T. E., Purcell R., Ogloff J. R. P. (2018). Sixty years of child-to-parent abuse research: What we know and where to go. Aggression and Violent Behavior.

[B61-ejihpe-15-00190] Stonard K. (2020). “Technology was designed for this”: Adolescents’ perceptions of the role and impact of the use of technology in cyber dating violence. Computers in Human Behavior.

[B62-ejihpe-15-00190] Straus M. A., Fauchier A. (2007). Manual for the dimensions of discipline inventory (DDI).

[B63-ejihpe-15-00190] Suárez-Relinque C., Del Moral-Arroyo G. (2023). Child-to-parent cyber violence: What is the next step?. Journal of Family Violence.

[B64-ejihpe-15-00190] Tian S., Zhang T., Chen X., Pan C.-W. (2021). Substance abuse and psychological distress among school-going adolescents in 41 low-income and middle-income countries. Journal of Affective Disorders.

[B65-ejihpe-15-00190] Van Dam D. S., van der Ven E., Velthorst E., Selten J. P., Morgan C., de Haan L. (2012). Childhood bullying and the association with psychosis in non-clinical and clinical samples: A review and meta-analysis. Psychological Medicine.

[B66-ejihpe-15-00190] Zych I., Ortega-Ruiz R., Del Rey R. (2015). Systematic review of theoretical studies on bullying and cyberbullying: Facts, knowledge, prevention and intervention. Aggression and Violent Behavior.

